# Prognostic stratification of renal cell carcinoma using a pathological triad of microvascular invasion, Fuhrman's grade and tumor size

**Published:** 2007

**Authors:** Gagan Prakash, Gagan Gautam

**Affiliations:** Department of Urology and Renal Transplant, Fortis Flt. Lt. Rajan Dhall Hospital, Sector B, Pocket 1, Aruna Asaf Ali Marg, Vasant Kunj, New Delhi - 110 070, India. E-mail: gagangg@gmail.com

## SUMMARY

In order to analyze the prognostic value of various clinico-pathological variables, the authors conducted a retrospective study on patients undergoing surgery for localized renal cell carcinoma (RCC).

Out of all the patients of RCC presenting to the authors' institution over a span of 15 years, those that underwent radical or partial nephrectomy were included in the study whereas those with metastatic disease at the time of diagnosis were excluded. The records of 230 patients who met the inclusion criterion were analyzed with respect to variables like clinical presentation, histological type, Fuhrman grade, tumor size, lymph node involvement and presence of microvascular invasion (MVI). The correlation of each of these variables with survival rates and recurrence was estimated. The overall cancer-specific mortality rate was 13% (31 of 230) and recurrence rate was 17% (39 of 230) on a median follow-up of 48 months (range three to 140). On multivariate analysis, microvascular tumor invasion, tumor grade and tumor size had the most significant correlation with disease-free survival and recurrence.

Using these three independent variables, survival probabilities were stratified into low risk (Fuhrman's Grade 1 or 2, diameter 7 cm or less, MVI absent); high risk (Fuhrman's Grade 3 or 4, diameter greater than 7 cm, MVI present) and intermediate risk (1 or 2 high-risk variables). On Kaplan Meier analysis, these three categories had a disease-free survival rates of 94.7%, 56.8% and 13.1% and cancer-specific survival rates of 94.7%, 61.7% and 32.0% respectively.

## COMMENTS

Recent understanding of cellular processes governing tumor biology has led to the development of various novel treatment options in RCC. Sunitinib, Sorafenib and Axitinib are kinase inhibitors that inhibit the vascular endothelial growth factor (VEGF), platelet-derived growth factor (PDGF) and c-kit receptor tyrosine kinases. Bevacizumab is a monoclonal antibody that is directed against VEGF. Temsirolimus inhibits the mammalian target of rapamycin.[1] Survival benefits reported with Sorafenib and Sunitinib has led to their approval for advanced RCC by regulatory authorities.[2] With the advent of these promising drugs, prognostication of patients with renal cell carcinoma has gained immense importance since viable options for further treatment can be explored in high-risk groups. Furthermore, after undergoing nephrectomy, patients are very anxious to know the further course of their disease.

Until the recent past, tumor stage was considered to be the most important prognostic factor. Other variables like clinical manifestation, tumor size, Fuhrman's grade, lymph node involvement and tumor necrosis have also been seen to have significant correlation in various studies. Microvascular invasion (MVI) is rapidly attaining a distinct position in oncology. It is defined as the presence of cancer cells inside intratumor microvessels. Its role as a prognostic factor has already been established in penile cancers and it plays an important role in algorithms, which decide the need for lymph node dissection following penectomy.

Its impact on prognosis of RCC is an emerging topic of interest. In a recent study, the five-year survival after surgery for low-grade RCC was estimated to be 45% and 90% in the presence and absence of MVI respectively.[3] Microvascular invasion is definitely evolving as one of the most important independent prognostic parameters in RCC. However, it is for the first time in the present study that an attempt has been made to combine MVI with tumor grade and size to stratify RCC patients into different risk categories.

The median follow-up period in this study was 48 months (range three to 140). Studies with a longer follow-up would be required to authenticate the predictive value of these risk categories. Including this risk stratification in the armamentarium of prognostic tools would help physicians in counseling patients about the severity of their disease and also in enrolling patients for novel therapies.

## References

[1] Larkin JM, Chowdhury S, Gore ME (2007). Drug insight: Advances in renal cell carcinoma and the role of targeted therapies. Nat Clin Pract Oncol.

[2] Bellmunt J, Montagut C, Albiol S, Carles J, Maroto P, Orsola A (2007). Present strategies in the treatment of metastatic renal cell carcinoma: An update on molecular targeting agents. BJU Int.

[3] Madbouly K, Al-Qahtani SM, Ghazwani Y, Al-Shaibani S, Mansi MK (2007). Microvascular tumor invasion: Prognostic significance in low-stage renal cell carcinoma. Urology.

